# Effects of neck-exercise and health promotion on headache outcomes in office workers: secondary analysis of the NEXpro stepped wedge cluster randomised controlled trial

**DOI:** 10.1186/s10194-025-01963-y

**Published:** 2025-02-12

**Authors:** Markus J. Ernst, André Meichtry, Kerstin Luedtke, the NEXpro collaboration group, Markus J. Ernst, André Meichtry, Kerstin Luedtke, Andrea Aegerter, Aulona Ajeti, Marco Barbero, Beatrice Brunner, Samira Buob, Jon Cornwall, Yara Da Cruz, Manja Deforth, Oliver Distler, Julia Dratva, Holger Dressler, Tobias Egli, Achim Elfering, Irene Etzer-Hofer, Salome Felder, Ramona Furrer, David Gemperle, Michelle Gisler, Sandro Grob, Michelle Haas, Tabea Holzer, Delia Hug, Venerina Johnston, Sandro Klaus, Gina M. Kobelt, Hannu Luomajoki, Markus Melloh, Corinne Nicoletti, Seraina Niggli, Andrea Nüesch, Achim Nüssle, Kristina Ribeli, Salome Richard, Nadine Sax, Monika Schmid, Katja Schülke, Rebecca Siebeneicher, Gisela Sjøgaard, Lukas Staub, Seraina Störi, Thomas Volken, Josephine Wagner, Ellen Wartmann, Thomas Zweig, Deborah Falla, Deborah Falla

**Affiliations:** 1 Centre of Precision Rehabilitation for Spinal Pain, School of Sport, Exercise and Rehabilitation Sciences, University of Birmingham, Birmingham, UK; 2 Institute of Physiotherapy, School of Health Sciences, ZHAW Zurich University of Applied Sciences, Katharina-Sulzer Platz 9, Winterthur, 8401 Switzerland; 3 School of Health Professions, Berne University of Applied Sciences, Berne, Switzerland; 4 Institute of Health Sciences, Department of Physiotherapy, Universität zu Lübeck, Lübeck, Germany

**Keywords:** Headache, Neck exercise, Office work, Cluster RCT, Stepped wedge design

## Abstract

**Background:**

Headache conditions have a high prevalence worldwide. Office workers with high and demanding workload, but low physical activity levels are considered vulnerable for suffering from headache. This analysis examines whether exercise combined with health promotion at the workplace is effective for headache relief in office workers.

**Methods:**

This study reports the results of secondary outcomes of a stepped wedge cluster randomized controlled trial. Office workers (*n* = 120) were randomly assigned to a twelve-week supervised intervention period, consisting of neck and shoulder girdle exercises with health promotion interventions performed at the workplace. Secondary outcomes were analysed and modelled for headache occurrence, frequency, and the Headache Impact Test-6 (HIT-6), accounting for possible effects for the intervention, the period it had been introduced, and interactional and nested effects.

**Results:**

At baseline, 88 of the 120 participants reported ≥ one headache episode in the past four weeks, with a mean headache frequency of 3.58 days for that period. The mean HIT-6 score for the entire cohort amounted to 53.6 points. For headache occurrence and HIT-6, the simplest model with the intervention only, showed the best statistical fit with an odds ratio for headache occurrence of 0.46 (95% confidence interval: 0.25 to 0.84), and − 2.23 (95% confidence interval: -3.35 to -1.12) points on the HIT-6 questionnaire. For headache frequency, the model accounting for interaction effects (intervention x period) had the best statistical fit and showed an incidence rate ratio of 0.57 (95% confidence interval: 0.44 to 0.74) for the first period, but not for later ones.

**Conclusions:**

Neck exercises and health promotion had a positive impact on headache occurrence, headache frequency and HIT-6, with the latter not reaching clinical importance. Although only statistically significant for headache frequency, larger effects were found during earlier periods or shorter interventional exposure for all outcomes, necessitating refresher sessions at later periods.

**Trial registration:**

NCT04169646.

**Supplementary Information:**

The online version contains supplementary material available at 10.1186/s10194-025-01963-y.

## Background

Headaches have the highest prevalence among neurological conditions worldwide [1, 2]. Neck pain is one the most frequent symptoms reported by patients with migraine and tension-type headache [3, 4], which are also the most frequent primary headache conditions [1, 2]. Sensitization and convergence of trigeminal and upper cervical afferences within the trigeminocervical complex may explain the occurrence of neck pain in primary headache conditions. Vice versa, it may also explain headaches as a symptom of neck conditions and hence provides a pathophysiological explanation for cervicogenic or post-traumatic headaches [5, 6].

Women in their early to mid-working ages are especially vulnerable to headache [7], as are office workers [8–12]. During office work, factors associated to headache and neck pain include long sedentary positions with decreased physical activity [13–16] due to high work-load [12], and when combined with cognitively stressful tasks [17, 18], mentally or emotionally stressful situations at the workplace, like lack of work autonomy, no personal development, working temporarily, or unpaid overtime [9, 19, 20]. Such working conditions are frequently associated with further unhealthy lifestyle factors [21, 22] such as sleep deprivation [23, 24] or a poor diet [25], which can lead to a vicious cycle of pain, inactivity and worsening of health.

The effectiveness of different non-pharmacological interventions for primary and secondary headaches have been examined in systematic reviews and meta-analyses, evaluating manual therapy [26, 27], physical or aerobic exercises [23, 28, 29] and psychological interventions [30, 31], but with only little to moderate effects on headache outcomes and with low level evidence [28–30]. Health promotion interventions, such as education have only recently been proposed [32, 33], and recent trials give insight on their additional effectiveness [34, 35]. However, for interventions implemented at the workplace, the level of evidence for either exercise or education remains low to very low [36]. Early trials focused more on exercise interventions [37–40], with later studies adding educational content, mostly focussing on posture correction and relaxation [41, 42].

The current randomized controlled trial combined an exercise and health promotion programme for office workers at their workplace [43]. To allow all participants to benefit from the intervention, while at the same time reducing the need for a larger sample size, a stepped wedge design was chosen [44, 45]. The current study reports on secondary outcomes, regarding the occurrence and frequency of headaches, and how quality of life might be affected by headache. The analyses of these outcomes is important, since headaches were present, in 73% of all participants at baseline, and in nearly 75% of those also reporting neck pain [46]. Specifically, the aim of this secondary analysis was to examine the effect of a combined specific exercise and health promotion programme, offered at the workplace, on headache outcomes in office workers with and without headache at baseline. We hypothesized that office workers would benefit from the intervention, by reporting less headache occurrence, less frequent headaches, and a reduced impact of headache on their daily life.

## Methods

**Fig. 1:**
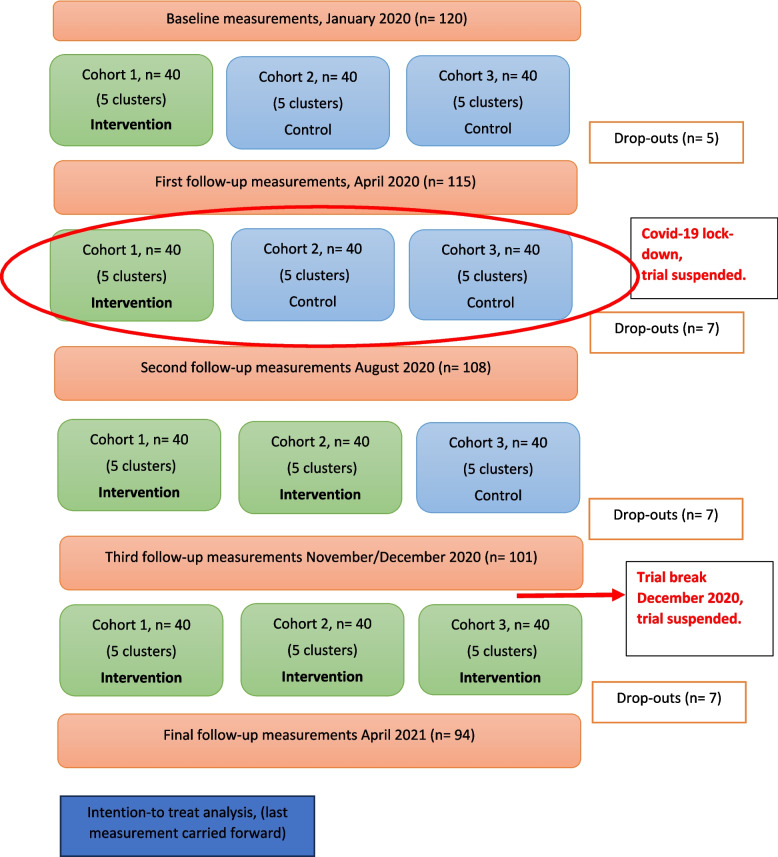
Flow chart of the study

This study reports the results of secondary outcomes of a cluster randomized controlled trial [43]. The original study used a stepped wedge design, meaning that at the end of the trial all participating office workers eventually received the intervention (see Fig. 1), which consisted of neck exercise, health promotion and workplace ergonomics. Before the start of the trial, the study was approved by the Ethical Committee of the Canton Zurich (Ref-No: 2019 − 01678), and it was additionally registered at clinicaltrials.gov (NCT04169646). All participants gave written informed consent at the beginning of the study.

### Sample size calculation

A sample size of *n* = 120 has been determined for the primary outcome of the NEXpro trial “productivity loss due to neck pain” with an alpha error of 0.05, power of 80% and an attrition rate of 20%, based on a previous trial [47, 48]. Fifteen clusters with eight participants each and, initially four measurement time points, would have led to 480 observations (15 × 8 × 4 = 480) [47, 49].

### Participants

Participants either suffered from neck pain or headache conditions or wanted to take preventive action against their occurrences. They had to be between 18 and 65 years old, working for at least 25 h/week in mostly sedentary positions and did not report suffering from serious health conditions of the neck as defined by the European taskforce [50].

### Randomisation

Participants were randomised within clusters of eight participants by an independent statistician to one of three cohorts, that defined the time points when the intervention began. Until the beginning of the intervention, they remained in the control phase (See flow chart, Fig. 1) [47]. At each time point, five clusters started the intervention phase.

### Exercise program

In detail, the intervention period was 12 weeks in duration and involved neck and shoulder girdle exercises performed three times a week within their clusters and at the workplace, for 20 min each. The focus was on strength and endurance for the neck flexors and extensors. Training intensity was individually adapted by using the ten-repetitions maximum. Progress was assessed every three weeks and training intensity was adapted accordingly. Exercise adherence, during the 12-week supervised intervention phase, including additional voluntarily home exercise, was monitored via an app (Physitrack ^®^ London, UK). One group session per week was overseen by a specially trained physiotherapist. Details on the exercise program can be found as a supplemental file [47].

### Health promotion interventions

Health promotion interventions were provided as workshops once per week. Workshop contents consisted of health-related topics for neck pain and headache conditions, and included *coping with stress*,* the importance of relaxation*,* sleep*,* diet and nutrition*,* general physical activity*,* mental health*,* the influence of digital media on health*,* managing conflicts at work*,* maintenance of motivation and for continued exercising*,* resilience* and more [43]. Once during the first two weeks of the intervention period, each office worker was visited at their workplace by a trained health worker, helping to adjust, if necessary, their individual workstation according to and with the help of a checklist [51].

### Outcome measurements

Secondary outcomes, which the current study dealt with included *headache occurrence* (yes/ no), *headache frequency* (days) and the *short-form Headache-Impact Test* (HIT-6/), a six item questionnaire assessing the impact of headache on daily life, including the functional and emotional burden that headache can cause [52–55]. These outcomes were assessed at all follow-up time-points by referring to the past four weeks.

### Data analysis

Data analysis for headache outcome variables was performed as proposed by Nickless et al. [45] for stepped wedge cluster randomized controlled trials.

In general, for every outcome variable, four different generalized linear mixed models were fitted, (1) computing the time-averaged intervention effect only (Model one), (2) adjusting this time-averaged effect for study period (Model two), (3) accounting for both a step change in the response once the intervention was introduced and a change in the response over calendar time by adding a period-intervention interaction effect (Model three), (4) and finally examining the intervention effect when nested in exposure time (Model four). Binomial models with logit-Link were fitted for *headache occurrence*, Poisson models with log-Link for *headache frequency* and linear models for *HIT-6.* Random Intercepts for subject and subject-cluster interaction were added as random effects to account for the correlation structure.

Statistical fit for models of all three headache outcome variables (*headache occurrence*,* headache frequency and HIT-6*) were compared using the Akaike information criterion (AIC) and Bayesian Information criterion (BIC). Additionally, model comparison with likelihood ratio tests for the nested models one, two and three were examined, Data was analysed according to intention-to treat. For those who dropped out of the trial, the last available value was carried forward. For those that remained in the study but answered “no” to the question, of whether they had suffered from a headache in the last four weeks, a value of zero (0) for headache days and a HIT-6 score of 36 (corresponds to all six items scored with “never”) was imputed.

All analyses were performed using the R statistical software. The lme4 package was used for model fitting.

## Results

**Fig. 2:**
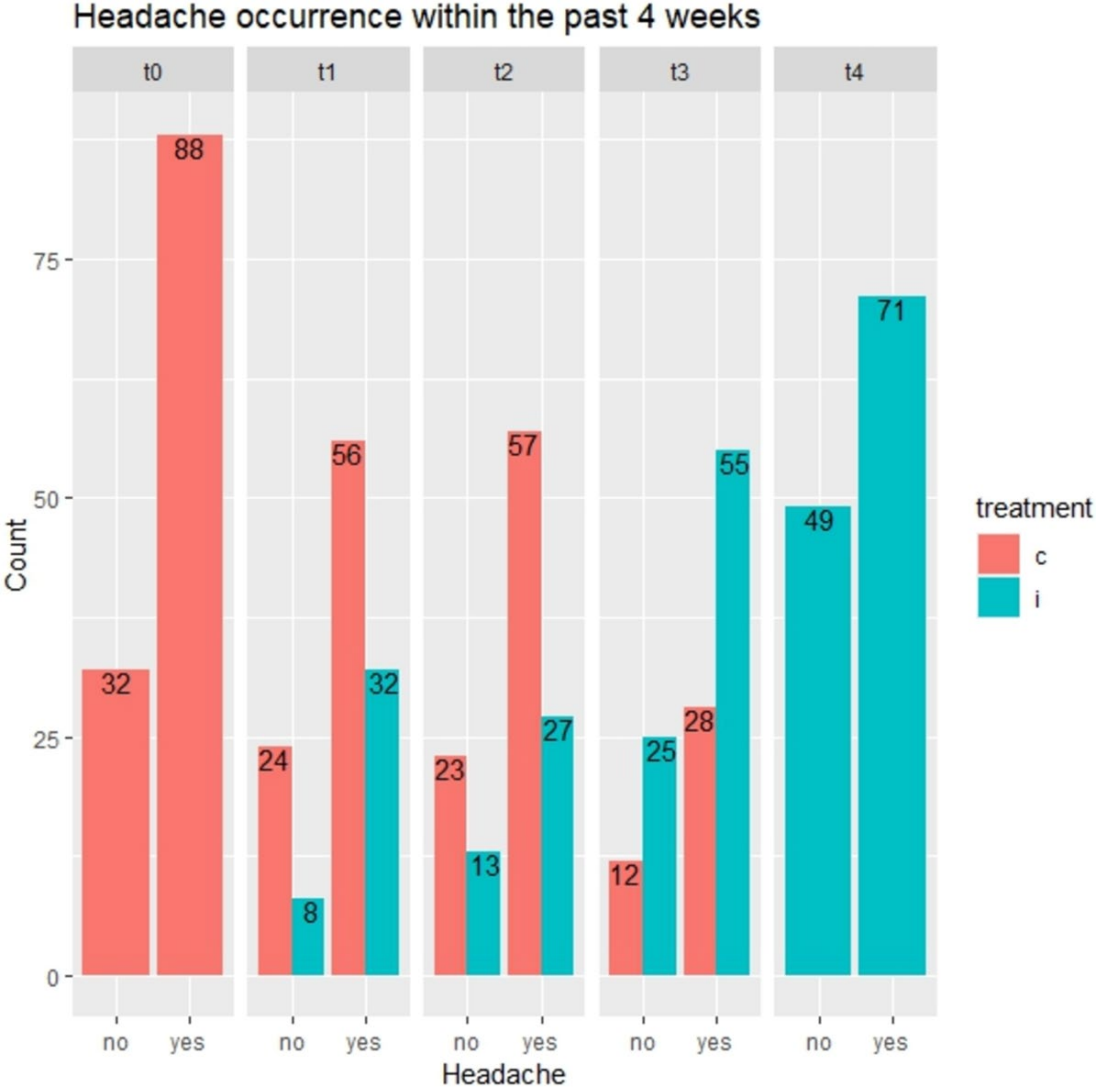
Bars indicate absolute frequency of headache occurrence, (no/yes) at five measurement time points= t0 for baseline to t4 for the final time point. The trial was suspended between t1 and t2 due to the pandemic, c= control in red, i= intervention in turquoise

**Fig. 3:**
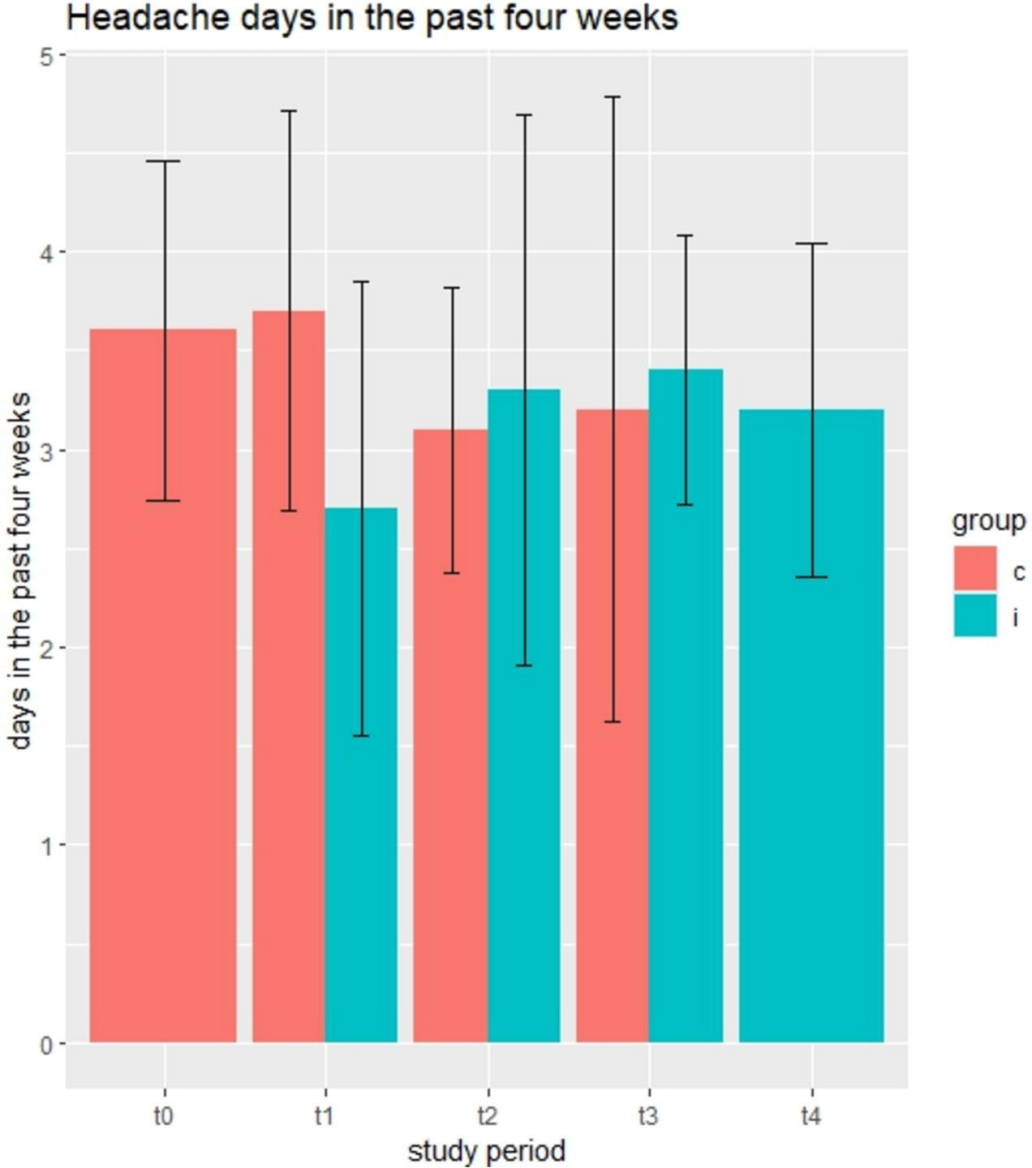
Mean (95% confidence intervals) headache days in the past four weeks) per group (c= control, i= intervention) and period (t= timepoint)

**Fig. 4:**
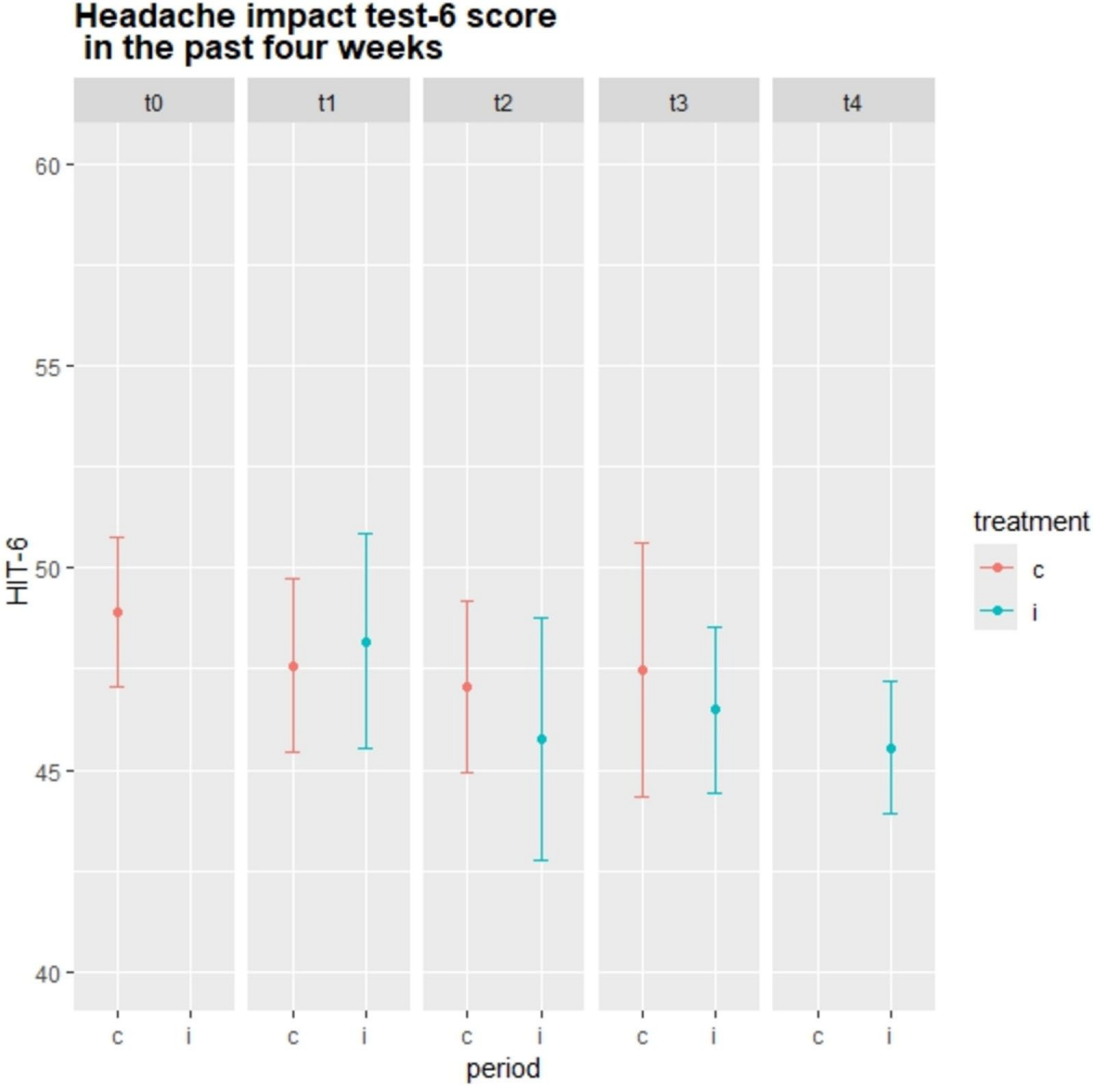
Mean (95% confidence interval) for Headache impact test (HIT) score per period (t= timepoint) and group (c= control, i= intervention). Values are mean and standard errors

In total, 120 (84 females) office workers participated in this trial. Further descriptives can be found in Table 1 or in related publications [46, 47]. Ninety-four participants (*n* = 94) completed the study, while 26 dropped out, most reported high workload situations (*n* = 8), job dismissal (*n* = 7) or pregnancy (*n* = 6) as reason for leaving the study. Another five (*n* = 5) reported various other reasons, including worsening health conditions (*n* = 2). Binominal models for headache *occurrence* are presented in and adjacent Table 2; Fig. 2, Poisson models for headache frequency are shown in and adjacent Table 3; Fig. 3 while linear models for the HIT-6 test are shown in and next to Table 4; Fig. 4.

**Table 1:** Baseline characteristics

Variable	Cohort 1	Cohort 2	Cohort 3
Participants (females)	40 (30)	40(26)	40 (30)
Age in years, mean (sd)	45.4 (10.7)	44.7 (9.3)	42.5 (9.4)
Number reporting ≥ 1 headache event/day in the last 4 weeks (%)	31 (77.5)	29 (72.5)	28 (70)
Number reporting ≥ 1neck pain event/day in the last 4 weeks (%)	33 (82.5)	27 (67.5)	35 (87.5)
Number of headache days in the last 4 weeks, mean (sd) (*n* = 88)	3.88 (6.08) (*n* = 31)	3.49 (4.70) (*n* = 29)	3.38 (3.48) (*n* = 28)
Number of neck pain days in the last 4 weeks, mean (sd) (*n* = 95)	6.58 (7.64) (*n* = 33)	6.48 (8.41) (*n* = 27)	7.29 (8.05) (*n* = 35)
Average headache intensity (0–10), mean (sd) (*n* = 88)	4.15 (1.99) (*n* = 31)	4.56 (2.41) (*n* = 29)	4.26 (2.08) (*n* = 28)
Average neck pain intensity (0–10), mean (sd) (*n* = 95)	3.13 (1.92) (*n* = 33)	2.88 (1.86)	2.93 (1.64)
HIT-6 score (36 to 78 points) (*n* = 88)	52.6 (7.1) (*n* = 31)	54.0 (9.5) (*n* = 29)	54.3 (7.1) (*n* = 28)
NDI score (0–50 points) (*n* = 95)	6.3 (4.9) (*n* = 33)	5.7 (5.9) (*n* = 27)	5.5 (4.0) (*n *= 35)
**Work-related variables**			
Level of employment < 50–100%, median (range)	80–89% (< 50 to 100%)	90–99% (50–59 to 100%)	100% (50–59 to 100%)
Time of employment (< 1 year to > 10 years), median (range)	6–10 years (< 1 to > 10 years)	3–5 years (< 1 to > 10 years)	3–5 years (< 1 to > 10 years)
Working with people in one room (1–2 to > 7 people), median, range	3–6 people (1–2 to > 7 people)	1–2 people (1–2 to > 7 people)	3–6 people (1–2 to > 7 people)
Average working time at the computer during working day in hours, median (range)	6–8 h (4–6 to > 8 h)	6–8 h (4–6 to > 8 h)	6–8 h (4–6 to > 8 h)
Self-perceived work performance (0 = worst to 10 = best possible), median (range)	9 (7 to 10)	9 (5 to 10)	8 (6 to 10)
Job stress index (JSI) category Total (n):	*N* = 40	*N* = 40	*N* = 40
More resources(n):	14	20	16
Balanced (n):	20	15	19
More stressors(n):	6	5	5

*HIT-6* Headache impact test score, *NDI* Neck disability index, *JSI* Job stress index, weighing job stressors against job resources [56]

**Table 2:** Binominal models for headache occurrence

Outcome Variable: Headache occurrence last four weeks				
Binominal model: Headache: Yes (1) or No (0)	Model 1 ~ Intervention	Model 2 ~ Intervention + Period	Model 3 ~ Intervention x Period	Model 4 ~ Period + Exposure time (Intervention nested in exposure time)
**Independent variable**	Odds ratio (95%CI)	Odds ratio (95%CI)	Odds ratio (95%CI)	Odds ratio (95%CI)
Constant	6.06 (3.15 to 11.67)	6.36 (2.94 to 13.77)	6.66 (3.01 to 14.74)	6.48 (2.96 to 14.17)
Intervention	**0.46 (0.25 to 0.84)**	0.98 (0.40 to 2.42)		-
Period 1		1.10 (0.48 to 2.50)	0.98 (0.40 to 2.35)	0.99 (0.43 to 2.28)
Period 2		0.76 (0.33 to 1.75)	0.94 (0.38 to 2.35)	0.88 (0.36 to 2.16)
Period 3		0.63 (0.23 to 1.73)	0.41 (0.12 to 1.39)	0.35 (0.12 to 1.05)
Period 4		0.32 (0.10 to 1.07)	0.28 (0.12 to 0.65)	0.24 (0.06 to 1.04)
Intervention x Period 1			1.38 (0.32 to 5.89)	
Intervention x Period 2			0.28 (0.0 to 1.14)	
Intervention x Period 3			1.67 (0.43 to 6.50)	
Exposure: 4 months				1.29 (0.49 to 3.37)
Exposure: 8 months				0.54 (0.15 to 1.87)
Exposure: 12 months				4.30 (0.75 to 24.71)
Exposure: 16 months				2.46 (0.34 to 18.00)
Observations	520	520	520	520
Log likelihood	−267.9	−265.1	−262.9	**−261.1**
Akaike Information Criterion (AIC)	**543.8**	546.2	545.7	544.3
Bayesian Information Criterion (BIC)	**560.8**	580.2	588.3	591.1

Values are odds ratios with 95 % Confidence intervals in brackets, all back transformed from logit scale. Figures in bold indicate the best fitting Model

Model one: the overall effect of the intervention had an odds ratio of 0.46 (95% confidence interval: 0.25 to 0.84)

Model two: By adding period to the model, no significant additional effect for any period was found. A trend for slightly larger effects, irrespective of the intervention, within later periods was found (See also Fig. 2)

Model three: No significant interaction for intervention and period was found. At period four when all participants had received the intervention, the intervention effect amounted to an odds ratio of 0.28 (95% CI: 0.12 to 0.65)

Statistical fit between models one, two and three was tested by ANOVA and demonstrated no better fit for Model two (*p*= 0.34) or three (*p*=0.11) compared to Model one

Model four: No significant effect for period and the intervention, nested in exposure time, was demonstrated. As was shown for Models two and three, a trend for a larger effect in later periods was found. AIC and BIC demonstrated a lower statistical fit, compared to other models and especially Model one

**Table 3:** Poisson models for headache frequency

Outcome Variable: Headache days in the past four weeks				
Poisson model: Incidence of headache events in the last four weeks	Model 1 ~ Intervention	Model 2 ~ Intervention + Period	Model 3 ~ Intervention x Period	Model 4 ~ Period + Exposure time (Intervention nested in exposure time)
**Independent variable**	Estimates (95%CI)	Estimates (95%CI)	Estimates (95%CI)	Estimates (95%CI)
Constant	1.97 (1.58 to 2.47)	2.04 1.61 to 2.57)	2.03 (1.61 to 2.57)	2.04 (1.62 to 2.58)
Intervention	0.84 (0.75 to 0.94)	0.78 (0.65 to 0.93)		-
Period 1		0.99 0.85 to 1.15)	1.08 (0.92 to 1.26)	0.96 (0.83 to 1.12)
Period 2		0.88 (0.75 to 1.04)	1.13 (0.95 to 1.34)	0.84 (0.71 to 1.00)
Period 3		1.03 0.85 to 1.24)	0.83 (0.65 to 1.05)	0.85 (0.69 to 1.07)
Period 4		1.12 (0.89 to 1.42)	0.87 (0.75 to 1.01)	0.90 (0.66 to 1.23)
Intervention x Period 1			**0.57 (0.44 to 0.74)**	
Intervention x Period 2			0.77 (0.58 to 1.01)	
Intervention x Period 3			1.07 (0.81 to 1.40)	
Exposure: 4 months				0.82 (0.67 to 1.00)
Exposure: 8 months				0.88 (0.67 to 1.14)
Exposure: 12 months				1.20 (0.87 to 1.67)
Exposure: 16 months				1.19 (0.78 to 1.81)
Observations	519	519	519	519
Log likelihood	−1091	−1088	**−1082**	−1083
Akaike Information Criterion (AIC)	2191	2192	**2184**	2184
Bayesian Information Criterion (BIC)	**2208**	2226	2226	2235

Estimates are incidence rate ratios (IRR) for headache events in the last four weeks, with 95% confidence intervals. Figures in bold indicate the best fitting Model

Model one demonstrated an IRR of 0.84 (95% CI: 0.75 to 0.94) in favour of the intervention

Model two indicated no period effect. A period-averaged IRR of 0.78 (95% CI: 0.65 to 0.93) in favour of the intervention was found

Model three showed a significant interaction between intervention and period, but no main effect for period (see also Fig. 3) After having received the intervention in period one, an IRR of 0.57 (95% CI: 0.44 to 0.74) in favour of the intervention was demonstrated

ANOVA showed a better statistical fit for Model three compared to Model one (*p*= 0.0021), also indicated by the AIC

Model four indicated a significant effect for period two with an IRR of 0.84 (95% CI: 0.71 to 1.00). The intervention nested in exposure time indicated better intervention effects after shorter periods, especially four months with an IRR of 0.82 (0.67 to 1.00)

**Table 4:** Linear models for headache impact test 6

Outcome Variable: Headache impact test 6 (HIT-6)				
Linear model Change in HIT-6 score.	Model 1 ~ Intervention	Model 2 ~ Intervention + Period	Model 3 ~ Intervention x Period	Model 4 ~ Period + Exposure time (Intervention nested in exposure time)
**Independent variable**	Estimates (95%CI)	Estimates (95%CI)	Estimates (95%CI)	Estimates (95%CI)
Intercept	48.11 (46.45 to 49.77)	48.81 (46.97 to 50.65)	48.81 (46.97 to 50.65)	48.81 (46.97 to 50.64)
Intervention	**−2.23 (−3.35 to −1.12)**	−1.07 (−2.83 to 0.69)		-
Period 1		−0.73 (−2.39 to 0.93)	−1.06 (−2.84 to 0.73)	−1.01 (−2.68 to 0.67)
Period 2		−1.89 (−3.54 to −0.23)	−1.58 (−3.36 to 0.20)	−1.66 (−3.42 to −0.10)
Period 3		−1.34 (−3.28 to 0.61)	−1.31 (−2.34 to 1.02)	−1.76 (−3.97 to 0.45)
Period 4		−2.25 (−4.60 to 0.10)	−2.20 (−5.23 to 0.83)	−2.91 (−5.88 to 0.06)
Intervention x Period 1			−0.09 (−2.77 to 2.58)	
Intervention x Period 2			−1.99 (−4.67 to 0.68)	
Intervention x Period 3			−1.12 (−3.74 to 1.50)	
Exposure: 4 months				0.24 (−2.19 to 1.70)
Exposure: 8 months				−1.75 (−4.26 to 0.77)
Exposure: 12 months				−0.63 (-−3.95 to 2.69)
Exposure: 16 months				0.77 (−3.31 to 4.86)
Observations	600	600	600	600
Log likelihood	−2066.0	−2063	−2062	**−2060**
Akaike Information Criterion (AIC)	**4141**	4143	4146	4145
Bayesian Information Criterion (BIC)	**4163**	4183	4194	4198

Estimates are mean differences with 95% confidence intervals. Figures in bold indicate the best fitting Model

Model one showed an average reduction on the HIT-6 scale of 2.23 (95% CI: -3.35 to -1.12) points after the intervention

Models two and three demonstrated effects by adding period to the model (Model two) or the interaction between intervention and period (Model three)

In Model two, the intervention showed the most precise and significant effect after period two. No interaction effect was found for Model three

The ANOVA did not demonstrate any better statistical fit for Model two (*p*= 0.18) or Model three (*p*= 0.52) compared to Model one

Model four: No effect of the intervention nested in time (exposure time) was found. Log likelihood indicated a better explanation than Models one to three, no ANOVA could be applied, as former Models are not part of Model four

## Discussion

The current study demonstrates that neck exercises and health promotion had a positive impact on *headache occurrence*,* headache frequency and headache impact*. Taking calendar and exposure time into account, the *headache occurrence* was reduced by more than 50%, the *headache frequency* (days in the last four weeks) was reduced by approximately 20%, and during the first intervention period by even more than 40%. However, the *impact of headache* on quality of life, work, cognition, and emotions was only marginally affected by the intervention. Slightly more than a two point reduction, on a scale from 36 to 78 points can be regarded a small effect not reaching clinical importance [57, 58]. Results for the HIT-6 might be partially due to low baseline values on the HIT-6, which were on average 53.6 points for those reporting headaches at baseline (Table 1), which implies only “some impact” on daily life by the headache [59]. Most outcome scores deteriorated after initial improvement, which is likely to be associated to the attrition rate of approximately 22%, and even more to the low programme adherence, with only 27% of participants following or even exceeding recommended exercise rates of three times per week, whereas 73% exercised less often, with 21% reporting weekly exercise fidelity of one or even less exercise sessions per week. Adherence was monitored only during the 12 weeks of supervised intervention and has been reported elsewhere [60]. The low adherence rates, which are comparable with similar trials [39, 61, 62], can severely limit the effectiveness of an exercise intervention. Methodological or psychosocial factors are reportedly responsible for a lack of adherence [63, 64]. While methodological factors have primarily been associated with the way adherence had been measured [63], psycho-social barriers for better exercise adherence include attitudes and beliefs towards treatment effectiveness or readiness to change behaviours like sleep habits, diets and managing or handling triggering factors including stress [64]. For the current study, only work-related prognostic variables were additionally sampled. The job-stress index (JSI) weighs stressors, like insecure employment, time pressure, excessive work demands, or harassment by colleagues or superiors against resources such as a holistic work with much work latitude and general acknowledgement by superiors or colleagues [56].Most participants of the current trial reported more resources than stressors (Table 1), which does not mean that no stress was reported, but that resources outweighed or at least balanced the reported stressors [56].

Stepped-wedge randomized controlled trials have several advantages over parallel controlled trial designs [44, 65]. Besides the need for smaller sample sizes, ethical issues are addressed since everyone will eventually receive the intervention [44, 65], which has specifically been stressed for interventions that have previously shown to be effective for specific health conditions, such as exercise and health promotion for neck pain, and for which it would be unethical to withhold them to those randomized to the control condition [48, 62]. Drawbacks of stepped-wedge randomized controlled trials are, however, a higher risk for attrition, especially if clusters are composed by a working population, and a job dismissal means also dropping out of the trial, and the lower sample size necessitates more elaborate statistical modelling for time effects [45, 66]. While the attrition rate remained low, exercise adherence has been modest, with only about half of participants were exercising twice a week at maximum [60]. While definitions for adequate adherence can vary among studies, Villanueva-Ruiz et al. recommended a 80% adherence rate [67] for neck-specific exercise to be effective, which has not been achieved in the current study, nor in most predecessor trials [39–41, 61].

With respect to exposure time, shorter intervention times, such as four or eight months showed better effects, especially for *headache frequency*, but partially for other outcomes too, while those that had been “exposed” longer to the intervention, but not any longer supervised, often showed reversed effects with time, making further nonadherence to the exercise and/or educational aspects of the interventions even more likely. Another time effect, often regarded important in stepped wedged designs trials, like seasonal or calendar time had no influence on headache outcomes, as can be seen in the Model two for both *headache occurrence* and *HIT-6* (Tables 2 and 4) indicating a nearly steady increase of the intervention effect with calendar time, which can be explained by the steady increase of participants having received the intervention while the size of the control group shrank.

The Covid-19 pandemic affected the current trial. In spring 2020, the first cohort needed to change to online exercise and health promotion meetings within their clusters. After the first follow-up at T1, the whole trial was suspended and restarted at the end of summer 2020, after the second follow-up T2 (see Flow chart, Fig. 1). For *headache frequency*, Model 3 indicates the largest effect at T1 (Table 3; Fig. 3), but mostly due to an increase of headache days for the second and third cohort that were, at that time still in the control condition, and only a slight decrease by the first cohort that had started the intervention. For the HIT-6, the largest intervention effect was found at time point 2, as indicated in Models 2–4. It can be speculated that during the early lock-down months, office workers that had already benefited from the intervention could keep up a more headache-friendly lifestyle, including regular exercising, which was partially reversed later, after the end of the lock-down, and by reintroducing the intervention to the other two cohorts. Similarly, Raffaeli et al. [68] found in their observational study that for the first lock-down month in Germany, headache patients showed a slight improvement in stress levels, sleep duration and medication use, but there was no change in their headache frequency or intensity. These effects mostly reversed in later lockdown months [68].

Exercise interventions have shown to be effective in headache conditions such as migraine [69, 70] and TTH [71, 72]. While general aerobic exercises have been recommended for both migraine and tension-type headache, both patient groups have shown a preference for local, dominantly passive interventions to their neck [70, 73], even though in some no long term improvements have been reported [74]. Few studies have examined the effects of health promotion in office workers with headache [41, 42]. Health promotion interventions, such as explaining pain mechanisms, or promoting self-efficacy, physical activity, sleep and diet strategies, or self-awareness have only recently been introduced [48]. Within this predecessor clinical trial, health promotion was compared with an exercise intervention for the neck and shoulder girdle in office workers to prevent or manage neck pain conditions [48, 62, 75]. Unfortunately [38, 41], the authors did not further examine or control for headache conditions [48, 62]. Although no overall between-group effects were found, subgroups with neck pain benefitted more from exercise in the short term (12 weeks), but not in the long term (12 months) [62], which, however corroborates the potential of health promotion interventions for a combined intervention program. In a systematic review by Lardon et al., [36], exercise or educational programmes at the workplace had been found to show only low or very low certainty of evidence on headache outcomes. None of the included trials in this systematic review used a combination of exercise and health promotion, as in the current trial. Meise et al. [35] examined the added effect of specific pain neuroscience education for patients with migraine, when compared to physiotherapeutic interventions such as manual mobilizations of the neck, strengthening of neck muscles, and postural and coordination exercises for the neck and head [35] The authors reported reduced migraine frequency for those who also received education. Their educational intervention was specifically tailored to migraine and may only partially be suitable for other headache conditions [35]. Compared to the trial by Meise et al., [35] the current trial focused more on general health promotion, that might fit for different pain conditions, including headache and neck pain. There was no specific focus on medication use or headache triggering factors [33], but sleep, diet, lifestyle, and relaxation were addressed [33, 35]. Those behavioural aspects in patient education, for better living with headache, was emphasized in a recent scoping review [76] compared to acquiring knowledge about the neurophysiology of headaches, with some portions of behavioural interventions also implemented within the health promotion part of the current trial.

### Strength and limitations

For most headache outcomes, the current study found treatment effects which are similar to those reported in comparable cluster randomized controlled trials for office workers [39–41, 61]. Headaches in the current trial were common and mostly more intense than neck pain (Table 1), which justifies the current analysis [46] and corroborates the need for better reporting and addressing headache comorbidities in future neck pain studies [4, 77, 78], especially as neck pain does not necessarily indicate a cervical origin [79]. In the current trial, headache occurrence was reduced to an acceptable level [55], while for headache frequency and the HIT-6, only minor and temporary effects were found.

A drawback of the current trial is that no proper headache diagnosis, according to the ICHD-3 criteria, was made [80]. There are several reasons for this limitation, first as this trial focused on a working population of office workers with, but also without headache or neck pain who were repeatedly re-assessed, we aimed to reduce the burden of further screening and assessment for all participants. Furthermore, the study did not include a headache specialist who could have made the diagnosis. Interventions at the workplace and in groups are not supposed to be as specific as individually tailored interventions with a health care provider. Furthermore, the current trial had a primary focus on productivity loss, including absenteeism and presenteeism by neck pain [47, 49]. These effects had unfortunately not been adjusted for accompanying headache conditions, that were largely prevalent (Table 1). Furthermore, more specifically and individually tailored exercises, including range of motion or motor control exercise, as well as health promotion for headache conditions might have led to better results [61, 81–83].

While attrition rate was acceptable and statistically accounted for by the intention to treat approach, adherence was not. Low headache and even lower neck pain disability values may also explain those low adherence rates, with participants potentially not seeing the necessity to perform exercises on a regular base [64]. Maintaining motivation to regular exercise was addressed in the health promotion sessions but frequent booster or refresher sessions may still be indicated and would need employers to provide time and space on-site for personnel.

To reduce the burden on participants, they were only asked to complete the questionnaires like the HIT-6, when they reported a headache occurrence in the past four weeks. This led to missing values, which were replaced by imputing the lowest possible total score value of 36 points.

## Conclusion

The current stepped wedge cluster randomized trial gives indications of the effectiveness of a combination of a regular neck-shoulder exercise with health promotion at the workplace and during working hours, on headache outcomes in office workers who mostly suffered from frequent headache conditions. While the (re-)occurrence and frequency of headaches have shown meaningful effects, especially with shorter exposure time periods, the impact and quality of life due to a headache condition, was minimally affected. Regular booster or refresher sessions might be indicated.

## Supplementary Information

Supplementary Material 1.

## Data Availability

No datasets were generated or analysed during the current study.

## Abbreviations

AIC: Akaike Information Criterion
ANOVA: Analysis of variances
BIC: Bayesian Information Criterion
HIT-6: Headache impact test-6 questionnaire
IRR: Incidence risk ratio
NEXpro: Neck exercise for productivity in office work
OR: Odds ratio
t: Time point
TTH: Tension type headache
