# Supplementary material for: Effects of neck-exercise and health promotion on headache outcomes in office workers: secondary analysis of the NEXpro stepped wedge cluster randomised controlled trial
Source: J Headache Pain. 2025 Feb 12;26(1):30. doi: 10.1186/s10194-025-01963-y (PMC11817237; doi:10.1186/s10194-025-01963-y)
Supplement: Supplementary file 1 — Supplementary Material 1. [file 10194_2025_1963_MOESM1_ESM.pdf]

Neck and shoulder exercises used in the NEXpro stepped wedge randomised controlled trial.

**Region**	**Aim**	
	**Strengthening**	**Stretching and relaxation**
**Neck region**	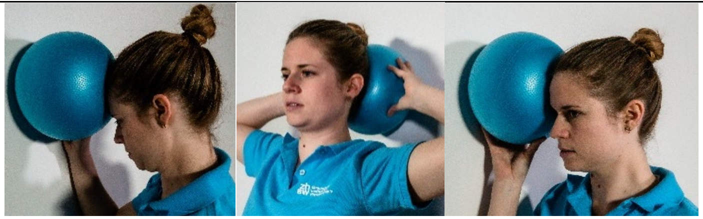<br>For Flexion, Extension and Rotation direction, either isometric contractions, or contractions within a small range of motions were used. Alternative to a soft ball, tera-bands could be used, or even pushing in one's own hand.<br>**Repetitions**: approximately **5 x for 5 seconds**	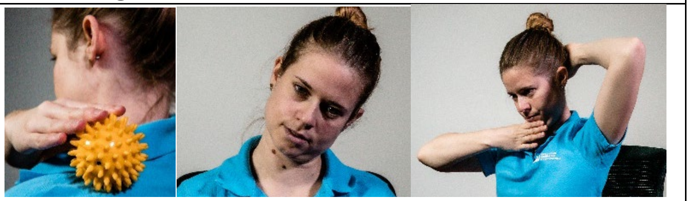<br>Self-massage and stretching the upper trapezius and upper cervical extensors were instructed.<br>**Repetitions**: approximately 3 times for 20 seconds.<br>Other balls, like a tennis ball could have been used.
**Shoulder (girdle) region**	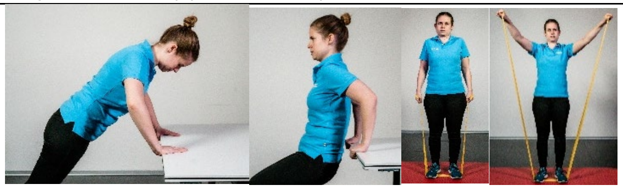<br>Frontal and backwards pushes at their own desk, shoulder-girdle shrugs and arm raises with a tera band and an extra push for shoulder girdle elevation had been instructed.<br>**Repetitions**: approximately 3 times 10 repetitions.	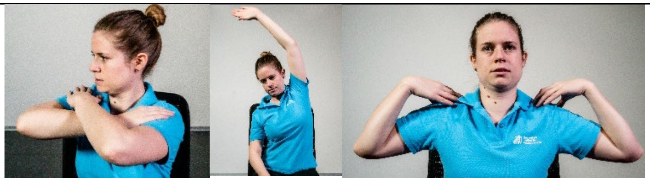<br>Upper body rotations, side bendings and shoulder-girdle circling had been instructed, preferably in standing position.<br>**Repetitions**: approximately 3 times for 20 seconds.

Individual adaptations could have been applied, if needed and indicated.

Ernst et al. Effects of neck-exercise and health promotion on headache outcomes in office workers: Secondary analysis of the NEXpro stepped wedge cluster randomised controlled trial, The Journal of headache and pain 2025.
